# Update on non-vector transmission of dengue: relevant studies with Zika and other flaviviruses

**DOI:** 10.1186/s40794-016-0032-y

**Published:** 2016-08-29

**Authors:** Lin H. Chen, Mary Elizabeth Wilson

**Affiliations:** 1 Travel Medicine Center, Mount Auburn Hospital, Cambridge, MA USA; 2 Faculty of Medicine, Harvard Medical School, Boston, MA USA; 3 Department of Global Health and Population, Harvard T.H. Chan School of Public Health, Boston, MA USA; 4 Department of Epidemiology and Biostatistics at University of California San Francisco, San Francisco, CA USA; 5 Division of Infectious Diseases, Mount Auburn Hospital, 330 Mount Auburn Street, Cambridge, MA 02138 USA

**Keywords:** Dengue, Non-vector transmission, Zika, Flavivirus, Mucocutaneous transmission

## Abstract

Human dengue virus infection without mosquito vector has been reported to occur as a result of mucocutaneous transmission, needlestick in patient care and laboratory accident, blood transfusion, bone marrow transplant, organ transplant, intrapartum and perinatal transmission, and breastfeeding. The emergence of Zika virus, another mosquito-borne flavivirus, has illustrated additional potential routes of non-vector transmission in humans. A recent study in another flavivirus, Japanese encephalitis virus, in pigs has also demonstrated non-vector transmission. We highlight some reports on dengue virus that have documented non-vector transmission and that are relevant to the transmission of Zika virus and other flaviviruses.

## Background

Dengue virus (DENV) is a flavivirus transmitted via the bite of the female *Aedes* mosquitoes, usually *Aedes aegypti* and *Aedes albopictus*. Rare cases of non-vector DENV transmission have been reported in the literature involving many routes including mucocutaneous exposure, needlestick in patient care and laboratory accident, blood transfusion, bone marrow transplant, organ transplant, intrapartum and perinatal transmission, and breastfeeding [1–3]. Until the Zika virus (ZIKV) outbreaks in the Americas led to the recognition of non-mosquito-borne transmission, most reports on flavivirus transmission via non-vector routes have been on DENV, with rare reports on other flaviviruses. We highlight some published reports and re-examine the potential of DENV transmission without a mosquito vector.

## Main text

### Non-vector transmission in DENV

Among the flaviviruses, DENV and yellow fever (YF) are known to cause hemorrhagic manifestations. Because of the higher incidence and much broader distribution of DENV, and its associated thrombocytopenia and hemorrhagic manifestations, DENV potentially has the greatest associated risk for direct or nosocomial transmission. Human DENV infection without mosquito vector has been documented primarily in case reports (Table 1). Several cases of DENV transmission via mucocutaneous or needlestick exposure were identified in non-endemic countries, all in patient care and laboratory settings [1, 4–8]. Of note, one case occurred in a healthcare worker that sustained a blood splash to face from a patient with confirmed DENV infection [1]. Because of the proximity of blood near mucous membranes and lack of percutaneous exposure, this case was considered to be a result of mucocutaneous transmission, while oronasal or aerosol transmission could not be proven.

**Table 1:** Summary of published cases of DENV transmission via non-vector routes

Route of transmission	Comment	References
Mucocutaneous	A health care worker who was splashed in the face by blood from a confirmed dengue patient was documented to have dengue infection, both identified to be DENV-3.	1
Percutaneous (needle stick, laboratory injury)	Health care workers including laboratory personnel acquired DENV infection after needlestick injuries.	4–8
Blood transfusion	Transfusion-transmitted dengue has been documented in Brazil and American Red Cross/CDC Dengue Branch; a DENV-4 outbreak in Brazil resulted in transfusion transmission in about a third of recipients of RNA-positive donations.	9–11
Bone marrow transplant	Transmission of DENV-4 in a 6-year old child from Puerto Rico via bone marrow transplant led to a fatality.	12
Solid organ transplant	DENV transmission occurred from donor to recipient after living donor liver transplantation.	13
Intrapartum/perinatal	Newborns whose mothers had acute DENV infections in the peripartum period developed dengue infection, ranging from mild febrile illness with thrombocytopenia to severe manifestations.	14–26
	Case reports have documented intracerebral hemorrhage and fatality in infants.	
	Systematic reviews and meta-analysis found increase risk for miscarriage for women with dengue during pregnancy, preterm birth, and low birthweight.	18–19
Breast milk	A woman confirmed to have DENV infection postpartum breastfed on days 2–4 of her illness. The infant developed symptoms of dengue starting on day 4 of mother’s illness, was confirmed by PCR with high viral load in blood, and breast milk was positive by PCR and culture.	3
Oronasal	No confirmed transmission reported, but the case of mucocutaneous exposure raises possibility of oronasal infection.	1
Sexual	None reported	-

DENV transmission has also occurred via blood transfusion [9–11], bone marrow transplant [12], and solid organ transplant [13]. Despite the concern for transmission risk from patients with bleeding diathesis, these reported cases of DENV infection from non-vector transmission were not attributed to sources with hemorrhagic manifestations.

The effect of vertical transmission of DENV on infants is highly relevant given the concern resulting from the recognition of ZIKV to cause adverse fetal outcomes including microcephaly. Many cases of intrapartum and perinatal DENV transmission have been described but none have reported congenital abnormalities [14–17]. Systematic reviews to date have found an association of dengue infection during pregnancy with increased rates of preterm birth and low birth weight [18, 19]. One meta-analysis that included 6071 pregnant women, of whom 292 had DENV infection during pregnancy, found an odds ratio of 1 · 71 (95 % CI 1 · 06–2 · 76) for preterm birth and an odds ratio of 1 · 41 (95 % CI 0 · 90–2 · 21) for low birth weight in those with DENV infection. The study also identified an association with miscarriage (OR 3.51:95 % CI 1.15–10.77) [19].

Because vertical transmission of DENV usually occurred in endemic countries, confirmation faced challenges in areas where testing may not be readily available. For example, a case series from Sri Lanka of women with DENV infection during pregnancy did not document infection in most of the newborns [20]. In contrast, other cases of infants born to mothers with confirmed DENV infection just before delivery have illustrated intrapartum or perinatal transmission; the newborns typically developed febrile illnesses with thrombocytopenia [16, 18, 21–26]. Some series included women with hemorrhagic manifestations in the peripartum period [16, 21–23, 25]. They delivered infants who developed febrile illness confirmed to be DENV, but most infants recovered. Nevertheless, fatality has been reported in infants born to mothers with severe DENV infection [15], as well as maternal and fetal death, and miscarriage [20]. Thus, outcomes of vertical transmission of DENV in neonates ranged widely.

Most previous cases of intrapartum and perinatal transmission were presumed to be via placental transfer of virus. In 2012, a case in New Caledonia of vertical transmission occurred possibly via breastfeeding [3]. The mother had fever, anemia, and thrombocytopenia peripartum (2 days before delivery) that was later confirmed to be DENV infection [3]. The infant also developed fever and thrombocytopenia but recovered. Serial blood samples of the mother and infant and breast milk were positive for DENV by RT-PCR, but not the cord blood [3]. Viral loads in breast milk and mother’s blood on the same day were similar, and virus was cultured from breast milk, thus confirming the passage of DENV via breast milk [3]. This case report called attention to the potential role of breastfeeding in transmitting DENV, a possible mechanism in perinatal infections.

Lastly, some investigators have proposed the possibility of DENV transmission via aerosol route. This mechanism was suggested in 21 healthcare workers who were serologically confirmed to have nosocomial DENV and where there was no known needlestick or mucocutaneous exposure to DENV-infected blood [27]. To date, aerosol transmission of DENV in humans lacks confirmation.

### Non-vector transmission of ZIKV

The rapid spread of ZIKV infection has brought awareness about its transmission via non-vector routes and suggest areas of study needed for DENV. The most important non-vector route appears to be sexual contact. Such transmission associated with returning travelers has been documented in several countries [28–32]. In 2008, a case of probable male-to-female sexual transmission occurred in a Colorado couple where a male scientist returning from Senegal and his wife who did not travel both were confirmed to have ZIKV [33]. Another was an autochthonous case in Florence, Italy, 2014, where sexual transmission occurred between a man who returned from Thailand and his girlfriend who had not traveled [32]. More recently, male-to-male sexual transmission of ZIKV was confirmed in Texas between a traveler returning from Venezuela and his partner who had not traveled [29]. Additional cases in several countries that lack autochthonous ZIKV circulation are under investigation. [28] Importantly, semen from a Tahitian man with hematospermia tested positive for ZIKV and the virus was replicative [34]. Another report suggested the possibility of long ZIKV persistence in semen in a 68-year-old man who was diagnosed with ZIKV infection in 2014 after returning to the UK from Cook Islands; his convalescent phase semen was positive for ZIKV by real-time reverse transcription PCR at 27 and 62 days after symptoms onset [35]. Belgian investigators that followed 4 ZIKV-infected men have also reported persistence of ZIKV RNA in semen up to days 56 and 68 in two of the men [36]. Such information will contribute to the basis of recommendations for ZIKV prevention.

A notable report raised the possibility of transmission through oral (saliva) or other body fluids besides semen in a woman living in Paris who had not traveled to any area with ZIKV circulation. She had sexual contact, both vaginal and oral, several times with a man who had been in Brazil and who had symptoms of ZIKV that resolved a day before their first contact [30]. In the woman, urine and saliva were positive for ZIKV RNA; her serum PCR was negative for ZIKV RNA but IgM was positive; vaginal swab was negative for ZIKV RNA. The index patient had positive urine for ZIKV RNA but plasma and saliva were negative; his semen culture grew ZIKV on days 18 and 24 [30]. Although ZIKV has previously been identified in saliva, [37, 38] including cultivable virus [38], transmission via saliva has not been documented to date. A case of ZIKV infection following a monkey bite in Indonesia and diagnosed in Australia suggested that transmission may have occurred through saliva, but mosquito transmission could not be ruled out [39].

Other studies have also reported positive tests for ZIKV in saliva and urine with ZIKV typically persisting longer in semen, saliva, and urine than in blood [34, 38, 40]. Although transmission has not been definitively described as a result of saliva or urine, it is conceivable that infection can occur from exposure to any body fluids that have replicative ZIKV. Percutaneous exposure has resulted in ZIKV infection. Cases in laboratory setting have been documented as well as subcutaneous injection of ZIKV-containing suspension to a volunteer [41, 42]. Recently, a study on a woman infected with Zika virus has demonstrated the presence of ZIKV RNA in endocervical swab, cervical mucus, and genital swab [43]. The clinical relevance manifested in a separate case report of female to male transmission [44].

Persons infected with ZIKV but who are asymptomatic are potential sources for transfusion transmission of the virus. During the French Polynesia ZIKV outbreak, nearly 3 % of blood donations tested positive for ZIKV by PCR. Perinatal transmission of ZIKV has been identified [45]. Many studies now support the causality link between infection during pregnancy and subsequent congenital microcephaly and other developmental abnormalities [46–48]. Breast milk has also tested positive for ZIKV by PCR, although culture did not yield replicative ZIKV; transmission via breast milk has not been shown to date [45, 49].

### Non-vector transmission of other flaviviruses

Other flaviviruses related to DENV and ZIKV include Japanese encephalitis virus (JEV), Murray Valley encephalitis, St. Louis encephalitis, West Nile virus (WNV), and yellow fever (YF). Transmission of these viruses occurs nearly always via mosquito bites, although rare human cases of non-vector transmission have occurred for JEV, WNV, and YF [50]. Studies in animals have also established viral spread via oral, intragastric, intranasal/aerosol, percutaneous, cutaneous, and mucous membrane exposure [50].

A recent article described vector-free transmission and persistence of JEV in pigs [51]. JEV is known to have a cycle involving waterbirds and *Culex* mosquitoes and a cycle involving pigs as amplifying hosts. However, investigation of some JE outbreaks failed to detect virus in local mosquitoes [51], which raised the question of alternative routes of transmission. By placing sentinel pigs with intravenously infected pigs, the investigators found that intravenously-infected animals developed fever after 24 h that lasted for 4–5 days; viremia persisted 3 days. Sentinel pigs in contact with infected pigs demonstrated viremia for 2–4 days. Examination of organs of injection-infected pigs found positive viral RNA in the lymph nodes, the ileum, parts of the nasal cavity, the brain, with particularly high levels in the tonsils. Shedding of virus occurred as early as 2 days after needle infection; cell cultures were positive confirming presence of live virus in oronasal swabs. Animals infected by contact shed live virus 6–10 days after contact; most oronasal swabs became positive for viral RNA about 4–7 days after infection; virus was isolated from most swabs. Histopathological CNS lesions were demonstrated for both needle-infected and contact-infected pigs. Interestingly, viral RNA was still present in the tonsils at 25 days post injection after infection but not other organs.

In summary, pigs shed JEV in oronasal secretions and can be infected via the oronasal route. Whether infected by injection, close contact, or oronasal inoculation, clinical symptoms, virus tropism, and CNS histological lesions were similar. The tonsils were prominent sites of virus replication and JEV persisted there for ≥25 days despite high levels of neutralizing antibodies.

### Challenges in determining non-vector routes of transmission of DENV

Studies in animals on the transfer of DENV via aerosol or intranasal or oral route have not been done. One challenge has been the lack of adequate animal models for DENV. Non-human primates can be infected with DENV but do not become symptomatic [52]. A recently developed mouse model (AG129 mice) has contributed to some understanding of human infection with DENV [52], but there are no reports to date of AG129 mice developing DENV infection via direct contact or oronasal exposure with infected mice.

Clearly non-vector transmission of DENV has occurred, and many possible routes have been described. The overall burden of DENV infection from known non-vector transmission appears to be low. Primarily the non-vector routes involve percutaneous or mucocutaneous exposure to infectious blood, vertical transmission, and receipt of infected products for treatment. Many of these have also been reported with ZIKV in current outbreaks, and some have also been described with other flaviviruses such as WNV and YFV [50]. For instance, vaccine strain of YFV has been transmitted via breastmilk resulting in febrile illness and meningoencephalitis in the infants [53–55]. Good infection control is the key to controlling DENV via non-vector transmission.

Unlike the ZIKV reports that document sexual transmission and the JEV study in pigs that confirmed oronasal transmission, DENV infection has not been attributed specifically to these routes of transmission. One case report of the blood splash to face that led to confirmed DENV infection was considered mucocutaneous transmission, but oronasal transmission might have been plausible. Possibly, oronasal and sexual routes of exposure can lead to DENV transmission, but are difficult to distinguish from mosquito-borne transmission in endemic countries where mosquito vectors are widely present.

It is also possible that routes of non-vector transmission of flaviviruses differ from each other. For DENV and ZIKV, infections in pregnant women clearly lead to different birth outcomes. While DENV in pregnant women has been associated with low birth weight infants and preterm birth, no congenital abnormality has been found, but detailed prospective studies are lacking. On the other hand, ZIKV acquired in utero has the much more severe consequence of microcephaly and multiple other abnormalities.

### Current knowledge gaps with respect to DENV non-vector transmission and public health implications of possible route of transmission

Many knowledge gaps in DENV non-vector transmission remain. Key questions that need exploration in view of discoveries from ZIKV include:

- Are there additional routes of DENV transmission, including oral, intragastric, intranasal/aerosol, and sexual routes?
- Is DENV present in body fluids other than blood and breast milk (urine, semen, vaginal secretions, saliva, tears), and what are the virus kinetics in these body fluids?
- If DENV is present in other body fluids, how long does it persist?
- What is the potential of DENV transmission via sexual contact?
- Is there any possible association of vertical transmission of DENV with congenital abnormalities?
- Is it possible for DENV transmission via breastfeeding to cause significant harm in infants?
- In dengue-endemic countries where mosquitos are ubiquitous, how will it be possible to determine whether a case of DENV is acquired via a mosquito bite or a non-mosquito route?

## Conclusion

In conclusion, documenting and confirming non-vector routes of transmission of DENV faces challenges. *Aedes* mosquitoes, the vectors of disease, are ubiquitous in most countries endemic for DENV and bites occur despite preventive measures; therefore vector-borne transmission is implicated foremost as the exposure. Non-vector transmission is more likely to be suspected in non-endemic countries that detect unusual infections with some link to returning traveler or unusual exposures. Studies on DENV transmission in animal models, like the study on JEV in pigs, would be helpful in our understanding of non-vector transmission of DENV. Similar studies will also be relevant in gaining deeper understanding of the pathogenesis and transmission of ZIKV.

## Abbreviations

DENV: Dengue virus
JEV: Japanese encephalitis virus
WNV: West Nile virus
YF: Yellow fever
ZIKV: Zika virus
